# The Importance of C4d in Biopsies of Kidney Transplant Recipients

**DOI:** 10.1155/2013/678180

**Published:** 2013-07-09

**Authors:** Rosana Rosa Miranda Corrêa, Juliana Reis Machado, Marcos Vinícius da Silva, Fernanda Rodrigues Helmo, Camila Souza Oliveira Guimarães, Laura Penna Rocha, Ana Carolina Guimarães Faleiros, Marlene Antônia dos Reis

**Affiliations:** ^1^Discipline of General Pathology, Nephropathology Service, Federal University of Triângulo Mineiro, Frei Paulino, 30, 38025-180 Uberaba, Brazil; ^2^Discipline of Immunology, Federal University of Triângulo Mineiro, Frei Paulino, 30, 38025-180 Uberaba, Brazil; ^3^Discipline of Cell Biology, Federal University of Triângulo Mineiro, Frei Paulino, 30, 38025-180 Uberaba, Brazil

## Abstract

Antibody-mediated rejection (AMR) is highly detrimental to the prolonged survival of transplanted kidneys. C4d has been regarded as a footprint of AMR tissue damage, and the introduction of C4d staining in daily clinical practice aroused an ever-increasing interest in the role of antibody-mediated mechanisms in allograft rejection. Despite the general acceptance of the usefulness of C4d in the identification of acute AMR, the data for C4d staining in chronic AMR is variable. The presence of C4d in the majority of the biopsies with features of chronic antibody-mediated rejection is reported, but this rejection without C4d staining is observed as well, suggesting that C4d is specific but not sensitive. Further studies on AMR with positive C4d staining in biopsy specimens are really important, as well as the study of novel routine markers that may participate in the pathogenesis of this process.

## 1. Introduction 

In renal transplant, the allograft is responsible for triggering many innate and adaptive immune mechanisms, either mediated by cells, such as macrophages and lymphocytes, or by soluble components, such as antibodies and the complement system, which can ultimately lead to graft rejection [1].

According to the Banff criteria [2], rejection may be mediated by cells or by antibodies and may be acute or chronic. Antibody-mediated rejection (AMR) is considered the main cause of kidney graft failure [3, 4]. The morphological diagnosis of AMR consists of various morphological changes together with C4d deposition in the microcirculation of the allograft. However, C4d deposition without AMR has been observed even in transplant glomerulopathy (TG), which is regarded as a chronic AMR and is characterized by proteinuria and loss of renal function over time, culminating in graft loss [5].

This review aimed to identify the role of C4d in episodes of AMR, especially in cases of TG.

## 2. Antibody-Mediated Rejection

Antibody-mediated rejection (AMR) is highly detrimental to the prolonged survival of transplanted kidneys, especially in highly sensitized patients, accounting for up to 30% of all posttransplant rejection episodes and resulting in 20–30% graft loss at 1 year if not treated successfully [6, 7].

Diagnosis of AMR requires the simultaneous presence of donor-specific antibodies, distinctive histopathological findings, and C4d deposition in peritubular capillaries (PTCs) [8]. Most centers that manage transplant recipients have incorporated routine C4d staining in the diagnostic pathology evaluation of all renal allograft biopsies [2, 9]. Capillaritis, glomerulitis, transplant glomerulopathy, and fibrosis/atrophy are concurrent histopathological AMR lesions and are associated with poor outcomes [10, 11].

In renal biopsy, AMR is characterized by the presence of acute tubular injury, peritubular capillaritis, glomerulitis, or arteritis, and it is immunopathologically characterized by C4d deposition in the peritubular capillaries of donor kidneys [9].

AMR may be of the following types: hyperacute, acute, and chronic. Hyperacute AMR occurs due to preformed donor-specific antibodies present in high titers, and it presents as graft failure that can occur within minutes or a few days after transplantation. The histopathology of hyperacute AMR is characterized by arteritis, interstitial edema, and severe cortical necrosis. Acute AMR is characterized by graft dysfunction manifesting over days, and it is a result of donor-specific antibodies, which may either be preexisting or develop *de novo* after transplantation. Histopathology in patients with acute AMR is also related to antibody-mediated endothelial injury, but it is less severe than the histopathology seen in hyperacute rejection. Moreover, biopsy often shows endothelial cell swelling, neutrophil infiltration of glomeruli and peritubular capillaries, fibrin thrombi, interstitial edema, hemorrhage, and positive C4d staining [7, 9, 12]. Chronic AMR, which is characteristically seen as transplant glomerulopathy in kidney biopsies, is characterized by glomerular mesangial expansion and capillary basement membrane duplication or splitting, interstitial fibrosis/tubular atrophy, and/or fibrous intimal thickening in arteries. Sometimes, peritubular capillary basement membrane multilayering is also observed on electron microscopy [7, 13, 14].

Due to donor-specific antibodies, AMR leads to the activation of the classical complement pathway, resulting in impaired graft function [15]. Circulating donor-specific antibodies produced by plasma cells bind to the endothelium of donor peritubular and glomerular capillaries and initiate the pathological sequence of AMR. C1q binds to the endothelium-binding donor-specific antibodies, thus initiating the classical complement pathway, a sequence which eventually leads to graft injury and dysfunction [16]. This anamnestic antibody response is typically directed to an endothelial MHC antigen [1]. Diagnosis is made by renal biopsy evidence of deposition of the split C4 complement component (C4d) in the peritubular capillaries, accompanied by morphological evidence of AMR, such as renal injury, allograft dysfunction, and the presence of donor-specific antibodies in plasma [1, 17].

In this study, we focused on the role of C4d in AMR. However, a combination of humoral rejection with cellular rejection, in which both may show complement deposition, is not unusual. This characteristic has been attributed to the low adhesion to immunosuppression protocols and/or to the quality of immunosuppression. The ideal treatment for mixed cellular and humoral rejections is unclear. Several studies report success using bortezomib [18] although these studies have mostly been uncontrolled; the use of eculizumab also has been reported in only one patient [19].

Rejection episodes humoral and cellular mixed are difficult to analyze because most occur months or years after transplantation, and biopsy may show acute and chronic injury [20]. In these cases, it is difficult to establish which branch of the immune system (cellular or humoral) was responsible for more damage [20]. It is known that positive immunostaining for C4d in peritubular capillaries combined with high levels of DSA in the serum suggests that AMR is the main cause of graft injury [18, 20]. On the other hand, low levels [20–22] and minimal immunostaining for C4d suggest that AMR is not the main cause of endothelial cell damage, particularly when the cellular rejection is well recognized [20]. 

A study from our group in patients with acute rejection showed that cases of cellular rejection have minimal immunostaining for C4d compared to cases of humoral rejection [23]. The minimal immunostaining for C4d in cellular rejection probably occurs through the activated via the mannose-binding lectin cascade [24, 25]; although complement factors activation on T-cell responses are mediated not only via specific signalling events in the T cell itself but also indirectly via alterations in antigen presenting cells and other cells that modulate T-cell activity [20]. 

Biopsies with evidence of AMR typically show microvascular inflammation. When found in the vascular endothelium, complement activation products such as C3a and C5a may have an influence on inflammatory response intensity, particularly in cells such as monocytes, natural killer (NK) cells, and CD8+ T cells [20].

A study using microarray analysis showed a high level of NK cells in biopsies of patients with chronic AMR and in patients with episodes of rejection mediated by cells with microcirculation injuries (peritubular capillaries, glomerulitis, and TG) [26].

## 3. Transplant Glomerulopathy

Transplant glomerulopathy (TG) is a lesion that has a primary immunological etiology [27], a specific morphology, and a strong association with humoral immunity [28]. The most likely explanation is that kidney transplants are essentially stable after recovering from the stress of implantation until specific diseases or conditions develop, including antibody-mediated rejection and recurrent renal diseases [29].

Originally classified as a variant of chronic allograft nephropathy of unknown etiology, TG is now recognized in patients with a previous history of humoral rejection, and it is associated with the deposition of the complement degradation product C4d, which suggests that TG may be one manifestation of antibody-mediated graft injury [2, 30–32].

TG is also associated with acute rejection and interstitial inflammation, so it is capable of causing progressive deterioration of kidney structure and function [33]. Moreover, it reflects a pathological process with severe negative implications for allograft survival [27], thus being associated with poor outcome.

Histopathologically, TG is characterized by duplication of the glomerular basement membranes, by mesangial matrix expansion, and by mesangial cell interposition [34, 35]. The early lesion consists of endothelial and mesangial cell swelling, with later mesangial matrix expansion, as well as eventual distortion and duplication of the glomerular capillary basement membranes. Interestingly, not only are these pathological changes isolated to the glomerulus but they also affect peritubular capillaries (PTCs) [36].

Studies have shown that ultrastructural changes in the endothelium are observed in glomerular endothelial cells and are accompanied by morphological abnormalities, such as cell vacuolation and widening of the subendothelial space, with the presence of electron-dense floccular material, probably due to thickening of the lamina rara interna [37, 38]. Furthermore, this thickening leads to projections that favor the formation of interdigitations and apical endothelial membrane vesicles, structures which, then, go into the capillary lumen [37, 39]. Therefore, such morphological changes trigger loss of glomerular endothelial cell fenestrae. Importantly, such changes were found in kidney biopsies performed within three months to one year after kidney transplantation [37, 40].

The changes regarding the lamina rara interna have an intimate relationship with the duplication of the glomerular basement membrane (GBM), because there is an association with mesangial cell interposition that culminates in an overall increase in GBM diameter at a rate of approximately 52.8 nm/year, with intense C4d deposition in peritubular capillaries (PTCs). In turn, mesangial matrix expansion and podocyte process deletion are observed later in TG, and the former is caused by increased mesangial matrix synthesis in the absence of cell proliferation [37].

A recent study demonstrated that the presence of one or more ultrastructural changes in glomerular TG was closely associated with the diagnosis of AMR, in the presence of either glomerulitis or peritubular capillaritis, as well as with C4d deposition in PTCs [40].

Even though the pathogenesis of TG is still unclear, researchers have hypothesized immune-mediated mechanisms with a strong emphasis on humoral immunity, for example, detection of donor-specific anti-HLA antibodies in the patients' serum [27, 41], and/or ± C4d staining of allograft biopsies [42]. Recent retrospective observational studies have raised new questions and suggested nonrejection-related diseases as possible causes in some patients [42].

Importantly, however, the morphological changes in TG are also found in other pathological conditions in kidney transplantation, such as recurrent ischemia, thrombotic microangiopathy, and membranoproliferative glomerulonephritis [43].

Membranoproliferative glomerulonephritis is an entity characterized by glomerular mesangial expansion caused by glomerular matrix expansion and an increase in cellularity. These structural changes are triggered by subendothelial and mesangial electron-dense deposits of immunoglobulin G (IgG) and complement component 3 (C3), leading to the duplication or splitting of the GBM [44].

In thrombotic microangiopathy, glomerulopathy is triggered by the activation of the coagulation cascade in renal tissue due to the presence of antiendothelial antibodies, C3 and/or C4 components, resulting in morphological changes similar to those found in TG and hemolytic-uremic syndrome (HUS) [43, 45, 46].

Ischemia/reperfusion injuries (IRI), the exacerbation of tissue damage upon reestablishment of circulation after a period of ischemia [47], contribute to the increase of immunogenicity, acute rejection, and chronic allograft dysfunction [48]. Ischemic tissue damage develops from impairment and subsequent cessation of blood flow, potentially beginning with brain death in the case of a deceased donor or with clamping of the renal artery in the procurement of living donor organs. During this time, the organ is subjected to hemodynamic instability, hormonal changes, and accumulation of toxic metabolic residue and depletion of adenosine triphosphate (ATP) [49].

The secretion of inflammatory mediators, such as IL-1, IL-6, and TNF, and expression of adhesion molecules by endothelial cell, permit the release of graft antigens and, consequently, complement system activation and recruitment of leukocytes. Thus, the immune response contributes to the enhancement of renal dysfunction after transplantation reperfusion through mechanisms of cellular self-injury [47, 50]. During IRI Complement C3 and/or C4 can be expressed by proximal tubular epithelial cells, glomerular epithelial cells, endothelial cells, and glomerular mesangial cells. Associated with leukocyte infiltration which triggers a local inflammatory response and consequently causes tissue injury [50]. The complement system seems to have an important role in ischemia/reperfusion injury. A possible trigger mechanism leading to activation of the lectin pathway is the binding of natural IgM to epitopes within ischemic tissue, with no involvement of previously implicated classical pathway activation. The alternative pathway is implicated in some instances, for example, in subsequent renal ischemia; it may either amplify C3 cleavage and the subsequent evolution of the injury, followed by initiation through the lectin pathway, or it may be seen as a separate event [50]. The involvement of the graft by these inflammatory events affects the reestablishment of renal function in the short and long time and may lead to rejection and reduced survival of renal transplant [49].

## 4. The Complement System and C4d

The complement system consists of several soluble or membrane proteins involved in both innate and adaptive immune responses, and it is the main effector component of adaptive humoral immunity. Its activation involves a cascade of sequential cleavage of proteins, yielding products with proteolytic activity, culminating in the generation of the key effector molecules of the complement system, such as opsonins C3b and iC3b, anaphylatoxins C3a and C5a, and the terminal membrane-attack complex (MAC) [51, 52]. The major functions of the complement system are to promote phagocytosis by opsonization of microorganisms and/or the host's own cells under certain conditions; to stimulate inflammation through the interaction of mediators of the complement system and receptors in phagocytic cells; and to cause direct cell lysis by forming the MAC [51–53]. Moreover, not only does the complement system comprise effector molecules that mediate its functions but it is also composed of several regulatory proteins and inhibitors that allow the body to regulate the location and intensity of activation of this system, preserving homeostasis [54]. In fact, although the complement system is primarily described as a system that fights infectious agents, its function as a promoter of tissue homeostasis has emerged. This is especially true in conditions of moderate activation, particularly neuroprotection, tissue regeneration, and removal of apoptotic cell debris [55–58].

The complement system can be primarily activated in three main ways: the classical, alternative, and lectin pathways. In general, the alternative pathway, which is phylogenetically older than the other pathways, is constantly and spontaneously initiated at the cell surfaces; this is particularly evident on microbial surfaces [53, 59, 60]. Similarly, the lectin pathway is also activated on cell surfaces rich in carbohydrates, such as those found in a wide variety of microorganisms, or after oxidative stress, such as in ischemia/reperfusion injury [61–65]. On the other hand, the classical pathway, which is phylogenetically more recent, is activated by antibody-antigen recognition and it is primarily associated with mechanisms of the adaptive immune response directed against either microorganisms or host cells, for instance, in autoimmune diseases or AMR [52, 66–69]. The complement system can also be activated by natural components, such as serum amyloid P or C-reactive protein. All these pathways lead to the formation of the so-called C3 convertases, which are similar in the classical and lectin pathways (C4bC2b) but different in the alternative pathway (C3bBb). These convertases cleave the C3 plasma protein into two fragments called C3a and C3b. C3a is an anaphylatoxin, and C3b is a part of the continuing activation of the complement system. Its association with any of the C3 convertases results in the C5 convertases, which act by cleaving the C5 component of the complement system into C5a and C5b, whereas C5a is a potent anaphylatoxin, C5b is a part of the final events of complement activation and it binds to C6, C7, C8, and C9 components, forming the membrane attack complex (MAC) [51–54]. Recently, a complement activation pathway that does not involve C3 convertases—in which the cleavage of C3 is triggered by the coagulation system—has been described [70, 71].

Once activated, the complement system does not discriminate between self- and foreign structures, and it is potentially damaging to both. Several molecules exert inhibitory or regulatory effects on the complement system. These substances can be soluble, such as factor I, factor H, C1INH, CFHR1, and FHL1, or bound to cell surfaces, such as CD46, CD55, CD59, and complement receptors (CRs) [53, 54, 72, 73]. One of the ways in which these regulators/inactivators act is by cleaving components generated by complement system activation, hence interrupting the formation of C3b by dissociating C3 convertases C3bBb and C4b2a. Alternatively, factor I can cleave C3b and C4b to their inactive forms (iC3b and iC4b). Recleavage of iC4b by factor I generates a soluble fragment called C4c and another surface-bound fragment called C4d; the latter covalently binds to the tissue and can, therefore remain at the site of complement activation [72–76]. Because the conversion of C4 into C4a and C4b is an event primarily related to the classical pathway (but also to the lectin pathway), the generation of C4d is related to antibody-mediated complement activation, for example, in humoral rejection (AMR).

C4b, the largest molecule from which C4d is derived, has an internal thioester that enables it to form a covalent bond with any free hydrogen group on target cells. When C4d is cleaved from C4b, the covalent bond between C4d and the tissue remains intact. Covalently bound C4d has a much longer half-life and, thus, remains at the site of complement activation, whereas antibodies bind to tissue by hydrostatic, van der Waals-type of interactions. The “footprint effect” of the internal thioester of C4d becomes strikingly apparent when the blood stream can clear all soluble/weakly bound molecules quickly, just like what happens with antibodies on the endothelial surfaces. Covalently bound C4d will not be affected, because it is anchored tightly to the tissue and, therefore, serves as a footprint of antibody-mediated rejection tissue injury [8, 41, 77] and has been regarded as “a magic marker” of complement activation and AMR tissue injury, because of its stability, its strong association with AMR, and its major impact on graft survival and patient treatment [8, 9, 16, 78, 79].

## 5. C4d and Antibody-Mediated Rejection

The introduction of C4d staining in daily clinical practice aroused an ever-increasing interest in the role of antibody-mediated mechanisms in allograft rejection [79]. The correlation between C4d deposition and graft survival was first reported in 1993 [80] and later confirmed by other studies [14, 81]. It was demonstrated that patients with suspected antibody-mediated injury in the renal graft had a linear C4d staining pattern in peritubular capillaries and that the presence of C4d was associated with impaired graft function [8, 82].

These studies led to general acceptance of the utility of C4d in the identification of acute AMR. In 2003, C4d was incorporated in the Banff classification [9], and the diagnosis of antibody-mediated rejection in renal allografts is currently based on criteria established in the 2007 Banff conference. The criteria also include morphological evidence of acute or chronic tissue injury, immunopathological staining for C4d in peritubular capillaries, and presence of circulating antibodies to donor human lymphocyte antigen or other antigens expressed on donor endothelial cells [83].

Thus, detection of C4d by immunohistochemistry (IHC) or immunofluorescence (IF) methods was then implemented in most pathological anatomy laboratories [7, 80].

Recent data on C4d staining in chronic antibody-mediated rejection is variable [7]. Even though the presence of C4d in the majority of biopsies with features of chronic antibody-mediated rejection is related, rejection without C4d staining is also observed [84]. Some studies have found no correlation between transplant glomerulopathy and diffuse C4d, and many other studies showed no C4d positivity [27, 85–88], which suggests that C4d is specific but not sensitive. Cases of TG in the absence of anti-HLA antibodies [86] or positive diffuse C4d staining [86–88] raised the possibility that the pathogenesis of this disease involves alternative mechanisms. Protocol biopsy studies have demonstrated another important characteristic of C4d, which is variability of staining over time, suggesting a constant flux between states of positive to negative C4d. In these biopsies, microvascular inflammation was present in spite of the absence of C4d staining [89].

A possible new form of AMR, namely, C4d-negative AMR, has been described only in its chronic manifestation [88]. Alloantibodies themselves can alter the state of the endothelium in the absence of complement or other inflammatory cells, and antibodies can induce injury through interaction with leukocytes, such as natural killer cells, without complement as a mediator [8].

Some studies have shown several endothelial-associated transcripts in kidney transplants, expression related to the processes of endothelial cell activation, repair, and angiogenesis—which are well-known mechanisms of AMR– and this molecule was found to be higher in all types of rejection, but much higher in AMR. The majority of these cases with chronic AMR features had no C4d staining. There was also a strong correlation between elevated endothelial-associated transcripts and the presence of anti-HLA antibodies. That, along with the low sensitivity of C4d for chronic AMR, has given rise to the concept of “C4d-negative antibody-mediated rejection,” which appears to be at least as common as C4d-positive AMR and has similar poor prognosis in terms of graft survival [10, 90, 91]. A study, however, in a C4d-positive patient with acute renal allograft rejection might benefit from intensive therapy, potentially preventing the previously reported high graft failure rate [12].

A recent study on renal biopsies with AMR has shown that the combination of early C4d positivity and thrombotic microangiopathy confers a significant risk of graft loss when compared to the patients with C4d positivity without thrombotic microangiopathy [92]. These data demonstrate that the association of C4d staining with other morphological aspects has more prognostic value.

Recently, treatment of allograft recipients with eculizumab was reported to inhibit the cleavage of component C5 of the complement system [93, 94]. The use of this drug significantly decreased episodes of AMR in patients. This finding demonstrates that complement activation is critical for the development of AMR, although use of the drug did not affect the deposition of C4d in individuals who showed high levels of donor-specific alloantibodies [94].

## 6. Conclusion

Although it is established that, in cases of AMR, positive C4d staining may be associated with greater severity, this marker does not seem to be loyal to the definition of this entity. With the increasing use of C4d by transplant pathologists worldwide, several shortcomings of C4d have been identified, and C4d appears to be a less sensitive marker than initially thought [8]. Furthermore, molecular studies have provided insight suggestive of a complement-independent form of AMR or C4d-negative AMR, in which C4d is obviously not helpful as a diagnostic tool [11].

Based on our current understanding, we believe that a diagnosis should be best reported on the basis of such as tubulointerstitial, vascular, and glomerular histological changes, amended to the presence or absence of C4d [12]. Further studies on AMR with positive C4d staining in biopsy specimens are very important, as well as studies on new markers that may participate in the pathogenesis of this process. Perhaps the next Banff conference in 2013 might bring new information about C4d-negative renal injury in the diagnosis of rejection [2], once this marker is of extreme importance in the diagnosis of rejection.
